# Preventing Mislabeling: A Comparative Chromatographic Analysis for Classifying Medical and Industrial Cannabis

**DOI:** 10.3390/molecules28083552

**Published:** 2023-04-18

**Authors:** Julio Salazar-Bermeo, Bryan Moreno-Chamba, María Concepción Martínez-Madrid, Manuel Valero, Joaquín Rodrigo-García, Farah Hosseinian, Francisco Martín-Bermudo, Manuel Aguado, Rosa de la Torre, Nuria Martí, Domingo Saura

**Affiliations:** 1 IDiBE, Institute for R&D in Health Biotechnology of Elche, University Miguel Hernández of Elche, Avda. de la Universidad, 03202 Elche, Spain; julio.salazar@goumh.umh.es (J.S.-B.); bryan.morenoc@umh.es (B.M.-C.); c.martinez@umh.es (M.C.M.-M.); dsaura@umh.es (D.S.); 2 Mitra Sol Technologies S.L. Parque Científico y Empresarial UMH, Edificio Quorum III, Avda. de la Universidad, 03202 Elche, Spain; 3 Instituto de Ingeniería de Alimentos para el Desarrollo, Universitat Politècnica de València, Avenida Fausto Elio s/n, Edificio 8E, Acceso F Planta 0, 46022 Valencia, Spain; 4 Departament of Health Science, Institute of Biomedical Sciences, Autonomous University of Ciudad Juárez, Anillo Envolvente del PRONAF y Estocolmo s/n, Ciudad Juárez 32310, Mexico; 5 Institute of Biochemistry, Carleton University, 1125 Colonel by Drive, Ottawa, ON K1S 5B6, Canada; 6 Andalusian Center of Molecular Biology and Regenerative Medicine-CABIMER, Junta de Andalucía-University of Pablo de Olavide-CSIC, 41092 Seville, Spain; 7 CTAEX, National AgriFood Technological Center “Extremadura”, Carretera Villafranco-Balboa, Km 1.2, 06195 Badajoz, Spain

**Keywords:** cannabinoids, validation, gas chromatography (GC), HPLC, overlapping

## Abstract

Gas chromatography (GC) techniques for analyzing and determining the cannabinoid profile in cannabis (*Cannabis sativa* L.) are widely used in standard laboratories; however, these methods may mislabel the profile when used under rapid conditions. Our study aimed to highlight this problem and optimize GC column conditions and mass spectrometry (MS) parameters to accurately identify cannabinoids in both standards and forensic samples. The method was validated for linearity, selectivity, and precision. It was observed that when tetrahydrocannabinol (Δ9-THC) and cannabidiolic acid (CBD-A) were examined using rapid GC conditions, the resulting derivatives generated identical retention times. Wider chromatographic conditions were applied. The linear range for each compound ranged from 0.02 μg/mL to 37.50 μg/mL. The R^2^ values ranged from 0.996 to 0.999. The LOQ values ranged from 0.33 μg/mL to 5.83 μg/mL, and the LOD values ranged from 0.11 μg/mL to 1.92 μg/mL. The precision values ranged from 0.20% to 8.10% RSD. In addition, forensic samples were analyzed using liquid chromatography (HPLC-DAD) in an interlaboratory comparison test, with higher CBD and THC content than GC–MS determination (*p* < 0.05) in samples. Overall, this study highlights the importance of optimizing GC techniques to avoid mislabeling cannabinoids in cannabis samples.

## 1. Introduction

*Cannabis sativa* L. has become a widely cultivated plant and the focus of scientific research in recent years. The plant has a documented history of use for early cordage and textile production, traditional medicine, and religious rituals. Today, the inflorescences, roots, resins, and oils from *C. sativa* are being utilized for biomedical, recreational, meditational, and spiritual purposes [1,2,3]. The various applications of *C. sativa* have been well-documented in early Indian and Chinese communities, as well as in Egyptian, Greek, Roman, and American populations, particularly for its analgesic, anti-inflammatory, antiemetic, and anticonvulsant properties.

At least 70 of the over 400 identified cannabis constituents are phytocannabinoids, a class of substances contained in the *C. sativa* plant [4]. Δ9-tetrahydrocannabinol (Δ9-THC) and cannabidiol (CBD) are the two most well-known cannabinoids. The biologically active form of these compounds does not normally exist in the plant. For instance, they already exist as the acidic precursors Δ9-tetrahydrocannabinolic acid (Δ9-THC-A) and cannabidiolic acid (CBD-A) [4]. These acidic precursors in cannabis have to go through a process known as decarboxylation when they are subjected to heat, light, or other environmental variables. During this process, the carboxyl group is removed, and the cannabinoids are transformed into their active forms, such as Δ9-THC and CBD. Because Δ9-THC, Δ9-THC-A, CBD, and CBD-A have distinct chemical structures and chemical formulas, this can affect how they interact with the body and how analytical tools can identify them. Laboratories that analyze, develop, or validate cannabinoid assays must consider the cannabinoid forms that might be present in the material being tested. An appropriate approach is, when evaluating, either to take into account the amounts of both the acidic precursors and the active forms or only concentrate on the active forms based on the expected outcome of the test.

Given the reported research on the functional properties of the inflorescences of *C*. *sativa*, the plant is currently being studied for therapeutic treatments of various diseases. Metabolic disorders and neurodegenerative pathologies [5,6] are being evaluated using *C. sativa* cannabinoids due to the presence of the endocannabinoid system [7,8,9]. For these reasons, the pharmacology of Δ9-THC is possibly the most well-studied of all phytocannabinoids. CB1 and CB2 are the most well-studied endocannabinoid receptors for Δ9-THC, where it works as a partial agonist at submicromolar doses [10,11,12].

The reported mechanism of action and, thus, therapeutic effects of *C. sativa* use are dependent on its phytocannabinoid profile and content [5]. Δ9-THC-A and CBD-A, along with their decarboxylated forms of Δ9-THC and CBD, have been the major compounds of interest in analytical laboratory and forensic determination. Additionally, the inflorescences of *C. sativa* also contain a considerable number of other phytochemicals such as terpenes, flavonoids, stilbenes, fatty acids, alkaloids, and phenols [13]. Among these compounds, cannabinoids and terpenes are the most abundant, accounting for over five hundred identified compounds, which play a role in the mechanism of action and health-associated benefits of the different *Cannabis* chemotypes [5].

The variety of strength and composition among *Cannabis* strains or sources is a significant problem in the medical use of cannabinoids, especially for CBD-A and Δ9-THC. Cannabis potency is normally evaluated by the concentration of these molecules; however, studies have revealed that potency varies greatly amongst strains, even within the same cultivar [14]. As a result, the medicinal benefits of cannabis might be unpredictable and difficult to manage, particularly in conventional clinical settings. This highlights the need for more rigorous and standardized testing, labeling, and quality control methods to ensure cannabis’ constant strength and composition for medicinal use.

Although phytocannabinoids have demonstrated functional properties, the uncontrolled use of cannabis due to its psychoactive effects has led to a prohibition in most countries due to the presence of Δ9-THC. Hemp (used for industrial purposes) and Marijuana (used for drug-type purposes) are chemotypes of *Cannabis* that primarily differ in their Δ9-THC-A concentration or its decarboxylated form content (>0.20%, *w/w* for drug type) as per European and Spanish legislation [15]. Material that is registered and certified under these regulations can be legally grown, distributed, and processed. Failure to comply with these regulations can have significant economic, legal, and social consequences for hemp farmers.

Multiple analytical techniques for the determination of Δ9-THC-A are based on the application of chromatographic principles, with gas chromatography (GC) coupled to either a mass spectrometer (MS) or a flame ionization detector (FID) becoming the main analytical equipment used [16]. Given its potential for the analysis of complex mixtures, GC–MS is able to identify a wide range of compounds, including phytoconstituents and metabolites in plant extracts as well as complex samples [17,18,19]. Additionally, GC–MS has been used for the analysis of specific compounds in commercial formulations [20,21], increasing the importance of GC–MS in the analysis of complex biological and chemical samples. However, these methods may encounter issues such as overlapping peaks, problems with decarboxylation and isomerization effects caused by injection temperatures or after derivatization [22]. Additionally, the use of short chromatographic methods, while rapid, may not provide sufficient resolution to accurately quantify Δ9-THC-A and CBD-A. Therefore, conventional GC methods may not be suitable to accurately quantify Δ9-THC and CBD-A, and the need for more accurate and faster analytical techniques for the determination of these compounds is an area of ongoing research in the field.

Recent controversies have arisen in relation to the analytical techniques used by forensic laboratories due to the difficulties in differentiating hemp from marijuana [23]. Forensic laboratories are now tasked with distinguishing seized *Cannabis* samples as either legal hemp or illegal marijuana; however, a high number of forensic laboratories currently lack reliable extraction protocols and analytical methods for this purpose [22]. This has led to a significant social problem. In response, the National Institute of Justice (NIJ) awarded the National Institute of Standards and Technology (NIST) with the DJO-NIJ-20-RO-0009 award in 2021 [24] to provide forensic laboratories with the necessary analytical tools to confidently make these distinctions [25]. To this end, the Chemical Sciences Division (CSD) at NIST has been developing an integrated measurement services program for forensic and cannabis (hemp and marijuana) analysis, known as the Cannabis Quality Assurance Program (CannaQAP) [26]. The program includes three exercises in 2021 and 2022, which will cover the determination of cannabinoids (including total CBD and total THC) and other determinations such as moisture and toxic elements. Additionally, the Association of Official Analytical Chemists (AOAC) has responded to this challenge by convening experts and approving consensus methods for the analysis of cannabis and hemp through the Cannabis Analytical Science Program (AOAC-CASP) in March 2019 [27]. CASP provides a forum for the examination of the science of hemp and cannabis analysis and for the development and maintenance of cannabis methods. Furthermore, the American Society for Testing and Materials (ASTM) established Committee D37 on Cannabis in 2017 [28] to develop standards for cannabis, with a focus on achieving voluntary consensus on proficiency testing. Committee D37 develops hemp analytics testing programs with different laboratories worldwide.

Currently, there is a need for analytical methods for cannabinoid substances that are reliable to accurately differentiate between hemp and marijuana. This is important due to the legal implications that arise from confusion between the two. This study focuses on one potential source of error and aims to establish reliable analytical methods for differentiation. The study compares two GC–MS methods and high-performance liquid chromatography with diode array detection (HPLC-DAD) for separating Δ9-THC from CBD-A and for determining accurate quantification of Δ9-THC in *Cannabis* plant extracts. This research can help to address medical and legal issues to provide reliable methods for future differentiation of chemotypes, especially hemp and marijuana.

## 2. Results and Discussions

### 2.1. GC–MS Analysis

**Figure 1:**
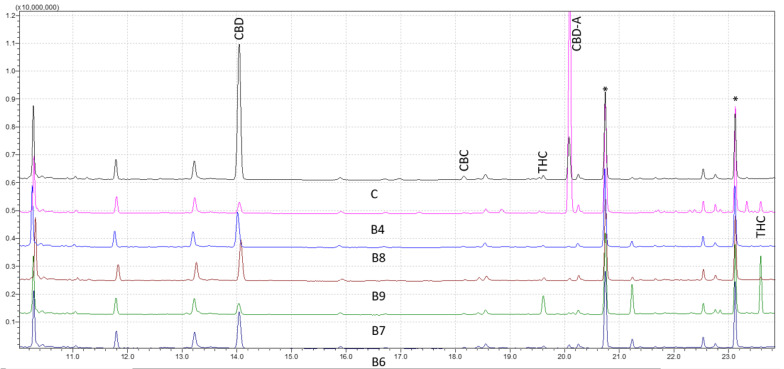
Chromatograms of five derivatized forensic samples analyzed through wide retention window (WRW) method by gas chromatography-mass spectrometry (GC–MS). Asterisks (*) represent trimethylsilyl esters (TMS) derivatives.

It has been proven that GC–MS is a potent analytical method for analyzing natural biocompounds, such as cannabis. The cannabinoids’ corresponding trimethylsilyl esters (TMS) were examined by GC–MS with electron ionization (EI) in this investigation. To describe and identify the retention times of various cannabis compounds, the total ion chromatograms of a solution containing the TMS derivatives were investigated. Figure 1 shows the results for forensic samples analyzed through the wide retention window (WRW) method. As seen, the peaks were likewise clearly separated, demonstrating the accuracy of the method in differentiating various cannabinoids. The studied cannabinoids’ retention times were shown to be 11.23 for cannabidivarin (CBDV) diTMS, 14.04 for CBD diTMS, 15.06 for tetrahydrocannabivarin (THCV) monoTMS, 17.54 for cannabigerol (CBG) diTMS, 18.18 for cannabichromene (CBC) diTMS, 19.64 for Δ9-THC diTMS, 21.26 for cannabinol (CBN) monoTMS, 20.09 for CBD-A diTMS, and 23.61 for Δ9-THC-A diTMS. No peaks were seen at the intended retention time area when a solvent blank and a solvent containing TMS derivatives were injected. This verified the high level of specificity of the method.

Analytical standards were used at nine various concentrations to validate the efficacy of the method. The outcomes were compared to samples taken from forensic cases with various cannabis contents (Figure 1). The reproducibility and stability of the retention periods were seen throughout several injections, which is a crucial sign of the robustness of the method. Overall, this work offers insightful information about the use of GC–MS for cannabinoids analysis. The findings point to the approach as a trustworthy and precise instrument for the identification and measurement of different cannabis components, which may have significant uses in both forensic and medicinal contexts.

**Figure 2:**
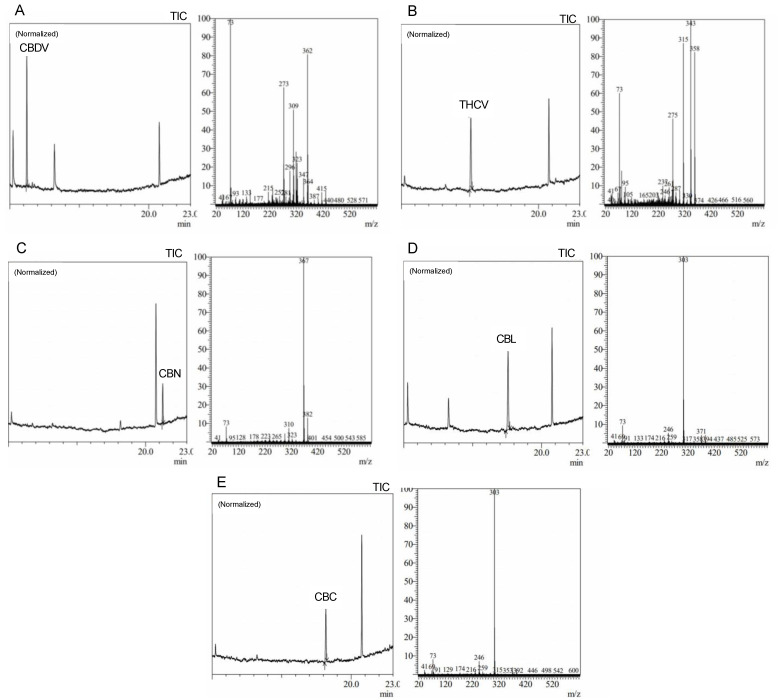
Average peak chromatograms and mass spectra of (**A**) cannabidivarin (CBDV), (**B**) tet-rahydrocannabivarin (THCV), (**C**) cannabinol (CBN), (**D**) cannabiciclol (CBL), and (**E**) canna-bichromene (CBC) derivatized cannabinoid standards.

Precise cannabis identification and quantification are critical for both medicinal and forensic uses, and MS is a potent tool for this assessment. In this study, we developed and standardized ion references for five different cannabinoids (Figure 2).

The results showed characteristic ion references (IR) of cannabinoids obtained by trimethylsilylation. CBD (IR: 390.25, 351.20, and 337.20), CBD-A (IR: 491.30, 559.30, and 492.30), CBC (IR: 303.20, 246.10, and 371.30), Δ9-THC (IR: 487.30 and 147.10), Δ9-THC-A (IR: 487.30, 413.20, 365.20, and 339.10), CBN (IR: 367.20, 382.20, and 310.10), and CBG (IR: 304.20, 246.10, and 383.30). These ion references were determined by carefully analyzing a range of standards and samples through MS and comparing them to previously published data and mass spectra libraries [29,30,31]. Our studies reveal the high quality and accuracy of our approach, with an average library match above 95%. Since it provides a reliable and exact approach to identifying and quantifying individual cannabinoids, this technology has significant implications for both research and practical uses in the area of cannabis analysis. We intend to contribute to the improvement of cannabis research and provide a helpful tool for researchers and analysts in this field by developing standardized ion references for a range of cannabinoids.

The linearity of the optimized GC–MS procedure was tested by evaluating a reference solution containing nine cannabinoids. Three independent replicates (n= 3) of the calibration curve at nine concentration levels were examined. The curves exhibited linear behavior within the concentration ranges examined, as shown in Table 1, with determination coefficients (R^2^) ranging from 0.996 to 0.999. The limits of quantification (LOQ) and limits of detection (LOD) were relatively low for all compounds, ranging from 0.33 to 5.83 μg/mL and from 0.11 to 1.92 μg/mL, respectively. This means the method can detect and quantify low concentrations of these compounds. CBD showed a lower LOD and LOQ than the other cannabinoids studied.

MS is a powerful analytical tool that offers superior selectivity and sensitivity compared to other detection methods, such as DAD and FID [32]. The GC–MS-based method developed in this study, the WRW method, represents a significant improvement in the analysis of cannabinoids. The method is highly selective, sensitive, accurate, and precise, as confirmed through rigorous validation according to the guidelines established by the AOAC. The linear range of the method was determined for nine cannabinoids, and the results showed a range between 1.20 and 37.50 μg/mL, except for CBD, which was between 0.02 and 37.50 μg/mL, with a resolution ≥ 1.50 between each cannabinoid evaluated, indicating the excellent selectivity of the method.

The precision of the method, evaluated as the percentage of relative standard deviation (%RSD), through inter-day precision ranged from 1.60% to 3.60% at the tested concentration solutions highlighting the robustness and accuracy of the method. In addition, the GC–MS experimental conditions were optimized to reduce the time of analysis while maintaining high resolution, with the best resolution results obtained by slowly reaching a temperature of 220 °C and increasing flow to 1.28 mL/min (Table 2).

These results demonstrate the high quality and robustness of the WRW method, making it a valuable tool for the accurate and precise analysis of cannabinoids in a range of samples, including forensic, medical, and environmental samples. The WRW method represents a significant advancement in the field of cannabinoid analysis and has the potential to improve the reliability and accuracy of cannabinoid analysis in a range of applications. With the present global legal status of cannabis and its potential medicinal effects, it is crucial to identify and quantify cannabinoids, especially Δ9-THC-A, Δ9-THC, CBD-A, and CBD, in hemp inflorescences.

**Figure 3:**
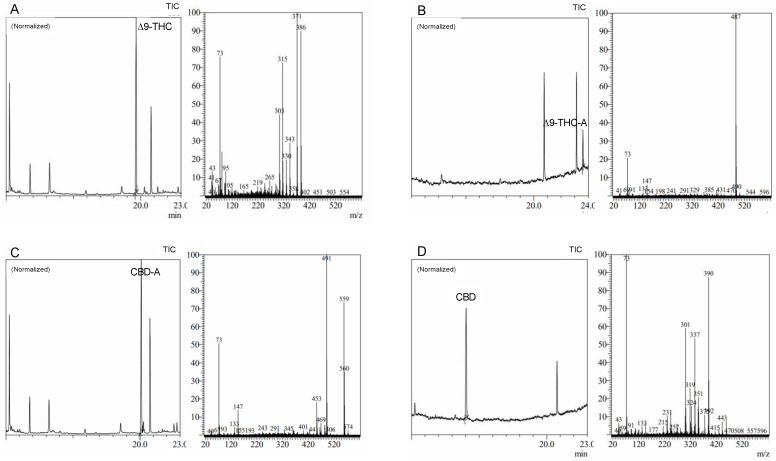
Chromatograms and mass spectra of (**A**) Δ9-tetrahydrocannabinol (Δ9-THC), (**B**) Δ9-tetrahydrocannabinolic acid (Δ9-THC-A), (**C**) cannabidiolic acid (CBD-A), and (**D**) cannabidiol (CBD) derivatized standards analyzed through wide retention window (WRW) method by gas chromatography and mass spectrometry (GC–MS).

The WRW method with MS detection was created to accomplish an accurate and reliable analysis of these compounds, offering the best resolution between these cannabinoids following derivatization (Figure 3). With main ions and ion patterns particular to each molecule, the WRW method demonstrated a high degree of specificity and selectivity. Moreover, a similar ion at 73 amu, a TMS derivative-specific signature, was seen in all MS spectra. When the MS spectra from standards and MS libraries were compared, a high degree of coincidence (up to 95%) was found, indicating the validity of the method. The WRW approach, which offers great resolution and discrimination of four cannabinoids, was also effectively used in the forensic examination of sample data. While cannabinoids were resolved well generally in chromatograms, Δ9-THC and CBD-A had the lowest resolution. Our findings emphasize the reliability and robustness of the method for the exact and accurate analysis of cannabinoids, which is essential for the creation of secure and efficient cannabis-based treatments.

**Figure 4:**
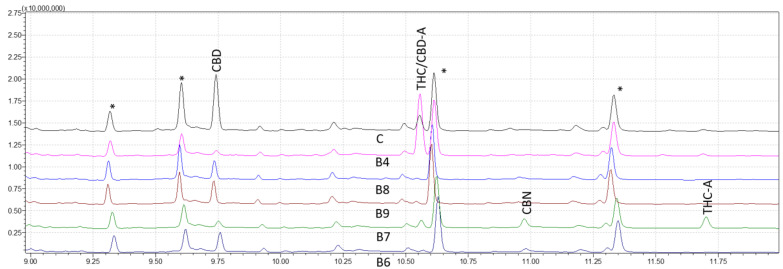
Chromatograms of five derivatized forensic samples analyzed through fast retention window (FRW) method of gas chromatography-mass spectrometry (GC–MS). Asterisks (*) represent trimethylsilyl esters (TMS) derivatives.

After establishing the analytical method validity using GC–MS, a shorter method named the fast retention window (FRW) method, developed and applied in GC-FID, was employed and adapted for this research. Figure 4 shows the chromatogram results of the FRW method, LODs and LOQs were determined, and interestingly, linear calibration curves were obtained to validate the FRW method for quantification; however, limitations of the FRW method appeared in this study as compared with the WRW method. A higher LOD, poor resolution between peaks, interferences coelution with the CBD-A peak and a non-reliability of the results invalidated the use of the FRW method for this study. The WRW approach, which offers great resolution and discrimination of nine cannabinoids, was also effectively used in the forensic examination of sample data. While cannabinoids were resolved well generally in chromatograms, Δ9-THC and CBD-A had the lowest resolution.

The approach of this study is critical in determining the presence and quantity of certain cannabinoids in hemp and marijuana samples. Because *Cannabis* plants are complex and contain a wide spectrum of cannabinoids, it is critical to have trustworthy testing procedures in place to ensure accurate findings. The use of MS detection in this investigation was very advantageous in this regard since it provides more selectivity and sensitivity than other detection methods, such as DAD and FID detection. The findings of our investigation demonstrated that the WRW approach was capable of achieving great resolution and sensitivity in the identification of nine distinct cannabinoids. In contrast, when reducing the analysis time, despite presenting similar limits of detection and quantification to the WRW method, the FRW method failed to provide adequate selectivity, with CBD-A and Δ9-THC displaying the same peaks and only being distinguishable through MS spectra analysis (Figure 4).

The development of a quick and effective technique for evaluating the presence and concentration of certain cannabinoids was made possible by the optimization of the GC laboratory analysis, which was another significant component of this work. This was accomplished by using various column oven settings and flow rates that were modified to produce the highest levels of resolution and selectivity. Overall, the findings of this study highlight the significance of having trustworthy analytical techniques in place for accurately identifying specific cannabinoids in hemp and marijuana samples, as well as the potential advantages of using MS detection coupled with GC analysis optimization in this context.

Multiple studies have proposed FRW methodologies for the detection of cannabinoids through GC [33], and plenty of them have reported an adequate resolution between cannabinoids. However, several limitations of the applications of FRW methods appeared in this study as compared with a WRW method. Other reports have also mentioned lower LODs, lower resolution, coeluting peaks, and the non-reliability of the results, which have prevented the use of GC for effective studies; for instance, GC-FID alone might not be a recommendable method for testing cannabinoids since itself cannot differentiate between molecules that coelute. Additionally, studies have reported similar drawbacks when applying GC-FID in food contaminants for TMS derivatives [34].

### 2.2. HPLC-DAD Analysis

In a cross-laboratory examination, the forensic samples were also examined using HPLC-DAD (Table 3). The calibration curves and correlation coefficients for the cannabinoids had been previously carried out and validated in compliance with the standards for underivatized cannabis samples established by the AOAC. The HPLC–DAD approach was accurate in terms of repeatability and intermediate accuracy. Particularly, it was observed that CBD reports from GC–MS varied by 14% from those obtained by HPLC-DAD, Δ9-THC GC–MS differed by 14% from the Δ9-THC HPLC-DAD data, and Δ9-THC-A GC–MS results differed by 15% from Δ9-THC-A HPLC-DAD.

The accurate determination of cannabinoid content in cannabis samples is critical for research, therapeutic development, and regulatory compliance. However, there is often significant variability in cannabinoid results between analytical methods and even within the same method when analyzing different samples. In this study, we analyzed five different samples (C, B6, B7, B8, and B9) using both GC–MS and HPLC–DAD to compare the variability in cannabinoid results. Interestingly, sample C showed similar results for CBD, CBG, CBN, and Δ9-THC-A, while Δ9-THC, CBC, and CBD-A showed the most variability, with an average of 23%. In sample B6, CBN and Δ9-THC showed similar results, while CBD, CBG, CBC, CBD-A, and Δ9-THC-A showed the most variability, with an average of 13%.

Sample B7 showed significant variability in its THC content between GC–MS and HPLC-DAD, with an average variability of 13% (*p* < 0.001). The results from samples C and B8 showed the most variability between GC–MS and HPLC–DAD, with an average variability of 13% of total CBD (*p* < 0.001), while sample B9 showed lower variability, with an average of 9% (*p* < 0.05). It is crucial to understand and account for these discrepancies between analytical methods, as both HPLC-DAD and GC–MS can have their own sources of error. Therefore, it is important to use accurate and validated analytical methods and carefully consider the potential sources of error when interpreting cannabinoid results.

The two most frequent instrumental techniques for cannabinoid analysis are GC–MS and HPLC–DAD. The heat from an ignition source or a GC injector, as well as time, decarboxylate the carboxylic acids and convert them to physiologically active forms. Derivatization as trimethylsilyl esters requires sample processing procedures. The most precise method for determining the native composition of the inflorescence is to employ a technique that does not require heat stress, such as HPLC–DAD, appropriate run times, and adequately resolved analytes of interest, especially those involved in legal issues.

In recent years, researchers have reported on the conversion of CBD to 9-THC. CBD has been found in studies to convert to THC under acidic circumstances [22]; also, according to a 2020 assessment of both in vitro and in vivo CBD conversion trials, these conversions did not occur in vivo [35]. Despite this, the possibility of misidentifying whether a sample contains CBD or THC remains, especially given the growing interest in CBD due to its non-psychoactive qualities and possible medicinal advantages. Several marijuana products on the market include a combination of CBD and THC, making it difficult for toxicologists to distinguish which chemical the sample contains. Laboratories utilize rapid GC conditions, potentially resulting in false positives for THC or under-reporting of CBD-A amounts. More research is needed to validate these findings and create better extraction processes that avoid the possibility of conversion and/or overlapping while still yielding more accurate cannabinoid results.

One of the work’s possible shortcomings is that it focuses primarily on GC–MS and HPLC-DAD procedures without considering alternative analytical techniques. Moreover, while the method was verified for linearity, selectivity, and precision in the research, the introduction of an internal standard might have enhanced the accuracy and precision of the results. To improve the validity of the findings, future research should consider using an internal standard to test the accuracy and precision of the analytical method.

## 3. Materials and Methods

### 3.1. Reagents

Certified standards of Δ9-tetrahydrocannabinolic acid (Δ9-THC-A), cannabidiolic acid (CBD-A), Δ9-tetrahydrocannabinol (Δ9-THC), cannabidiol (CBD), cannabinol (CBN), cannabigerol (CBG), cannabidivarin (CBDV), tetrahydrocannabivarin (THCV), and cannabichromene (CBC) were purchased from Cayman (Barcelona, Spain), Sigma-Aldrich (Madrid, Spain), and Cerilliant Corporation (Madrid, Spain). N,O-Bis(trimethylsilyl)trifluoroacetamide with trimethylchlorosilane (BSTFA:TMCS, 99:1, *v/v*), was supplied by Sigma-Aldrich (Taufkirchen, Germany). Millipore membrane filters (0.45 µm) were supplied by Merck (Darmstadt, Germany). Ethanol (200 and 190 Proof (100%) Non-Denatured Alcohol, ACS/USP grade) was obtained from Pharmco. Acetonitrile (LC-MS grade) and pyridine (certified ACS) were obtained from Fisher Scientific (Madrid, Spain).

### 3.2. Sample Preparation and Derivatization

Cannabis samples were collected in accordance with the UNODC recommended sampling method [36] and obtained from forensic cases. The material was submitted to our laboratory for routine testing. The dried material was ground and extracted with 99.8% ethanol using sonication for 15 min. The extract was then filtered using 0.45 μm filters and transferred to a GC vial for derivatization and to an HPLC vial for direct analysis. Prior to derivatization, GC vials containing samples and standards were evaporated to dryness using a Genevac miVac Duo concentrator at a temperature of 80 °C for 30 min. Derivatization was performed by adding equal amounts of BSTFA:TMCS and pyridine (100–200 µL) to the vials, capping, mixing, and incubating for 30 min at 80 °C before GC–MS analysis.

### 3.3. GC–MS Analysis

The determination of cannabinoids in forensic samples was performed using GC–MS, following a previously reported method by [37] and adapted by the research laboratory. The GC–MS instrument used was a Shimadzu QP 2010 Plus, equipped with a Restek Rxi-35Sil MS column (35% silphenylene) with 0.25 mm ID and 0.25 μm film thickness. Two methodologies were adapted and developed for this analysis: a WRW method where helium was used as the carrier gas (constant flow of 1–1.30 mL/min; linear velocity of 36.50 cm/s), and the oven temperature was ramped from 60 °C (30 s) to 220 °C at 25 °C/min (held for 10 min) and then to 300 °C (at 10 °C/min), where it was held for 15 min. The other method was a fast retention window (FRW) method of analysis previously described for GC-FID with a constant flow of 1.27 mL/min and linear velocity of 41.60 cm/s. The oven temperature was ramped from 80 °C (30 s) to 220 °C at 20 °C/min, to 260 °C (at 60 °C/min), and then to 300 °C (at 20 °C/min), where it was held for 5 min. The injector, interface, and ion source temperatures were set at 250, 280, and 220 °C, respectively, with a filament voltage of 70 eV. MS acquisition used the full scan mode with a mass range of 40–600 amu. For each analysis, 1 µL of the derivatized sample was automatically injected (AOC-20s automatic liquid injection system– autosampler, Kyoto, Japan) in splitless mode. Data acquisition, processing, and instrument control were performed using GC–MS Solution Software vs. 4.52 (Shimadzu Corporation).

The linearity, selectivity, accuracy, and sensitivity of the GC–MS technique were all validated. To assess retention times and retention windows, each cannabinoid was administered independently. The peak–area ratio of the standard was plotted against its concentration to create a linear regression curve for each constituent. The standard curves were used to compute the slope, y-intercept, and coefficient of determination (R^2^). LOD and LOQ were computed with a factor of 3.3 or 10, respectively, together with the standard deviation of the y-intercept and the slope of the linear curve. The selectivity of the method was evaluated by determining the resolution in total ion chromatogram (TIC) analysis for nearby cannabinoids with comparable fragment peaks. The allowable values for the resolution were ≥1.5. The accuracy of the method was assessed by calculating the inter-day %RSD based on sample injections over three consecutive days (n = 3).

### 3.4. HPLC-DAD Analysis

Filtered extracts were placed in HPLC vials and diluted 1:10 in 99.8% ethanol prior to being stored at 4 °C for HPLC analysis. The cannabinoids were separated using an Agilent series 1200 apparatus (Santa Clara, California, US.), which is coupled with a temperature-controlled autosampler, binary pump, and DAD. The separation was achieved on a Poroshell column 120 SB-C18, 2.7 µm, 4.6 × 150 mm. Under gradient conditions at 0.5 mL/min, the mobile phase compositions were (A) 0.1% formic acid in water and (B) 0.1% formic acid in acetonitrile. The gradient used to accomplish the separation was as follows: 0–8 min, 65% B; 8–12 min, 65–95% B; and 12–13 min, 95% B. After each run, a 5 min column re-equilibration was carried out. The injection volume was 2 μL, and quantitation was performed at 214 nm.

### 3.5. Statistical Analysis

A statistical analysis of cannabinoid content in Cannabis samples determined by both GC–MS and HPLC was performed using GraphPad Prism 8.0.2. software. The experiments were carried out in triplicate. A one-way ANOVA followed by multiple comparisons by the Bonferroni post hoc test. Statistical significative differences between cannabinoid content by both methods were considered when *p* < 0.05.

## 4. Conclusions

In conclusion, while GC is a cost-effective and efficient method for detecting cannabinoids, the resolution between cannabinoids can be affected when attempting to reduce analysis time. MS detection provides more selectivity and the opportunity to study ion fragmentation, but when evaluating derivatized samples, WRWs and better resolution techniques are crucial for reliable identification and distinction between cannabinoids. These findings are particularly significant for forensic investigations, dose-dependent trials, and legal matters involving the incorrect application or detection of cannabis. By addressing GC analysis mistakes that cause misunderstanding between CBD-A and Δ9-THC, trustworthy standard procedures for detecting cannabinoids can be established. However, for cannabinoid analysis of the native composition of the plant, HPLC–DAD might remain the most suitable method according to these results.

## Figures and Tables

**Table 1:** Method validation parameters.

Compound	Lineal Range (μg/mL)	Slope	y-Intercept	R^2^	LOQ (μg/mL)	LOD (μg/mL)	Precision (%RSD)
CBD	0.02–37.50	827,246.44	27,307.49	0.996	0.33	0.11	1.60
Δ9-THC	1.80–37.50	507,047.14	−1,416,117.14	0.999	5.83	1.92	1.72
CBDV	1.20–37.50	579,356.70	1,425,500.38	0.996	4.98	1.64	2.60
THCV	1.20–37.50	604,067.54	1,039,094.73	0.996	4.98	1.64	0.70
CBG	1.20–37.50	726,580.29	617,352.18	0.997	4.31	1.42	1.80
CBC	1.20–37.50	537,492.31	1,034,974.02	0.998	3.51	1.16	0.20
CBN	1.20–37.50	476,825.07	1,449,121.90	0.997	4.31	1.42	0.90
Δ9-THC-A	1.20–37.50	389,534.97	549,968.28	0.998	3.51	1.16	3.60
CBD-A	1.20–37.50	389,534.97	549,968.28	0.998	3.51	1.16	8.10

Cannabidiol, CBD; Δ9-tetrahydrocannabinol, Δ9-THC; cannabidivarin, CBDV; tetrahydrocannabivarin, THCV; cannabigerol, CBG; cannabichromene, CBC; cannabinol, CBN; Δ9-tetrahydrocannabinolic acid, Δ9-THC-A; cannabidiolic acid, CBD-A; coefficient of determination, R^2^; limit of quantification, LOQ; limit of detection, LOD; relative standar deviation, %RSD.

**Table 2:** Gas chromatography (GC) temperature gradient program parameters in the wide retention window (WRW) method.

	Rate (°C/min)	Value (°C)	Hold Time (min)	Run Time (min)
Initial	0.00	60.00	0.50	0.50
Ramp 1	25.00	220.00	10.00	16.90
Ramp 2	10.00	300.00	15.00	39.90

**Table 3:** Gas chromatography-mass spectrometry (GC–MS) vs. high-performance liquid chromatography–diode array detection (HPLC–DAD) quantification of cannabinoids (%) from same cannabis samples.

Sample	Method	CBD	CBG	CBN	Δ9-THC	CBC	CBD-A	Total CBD	Δ9-THC-A	Total THC
C	HPLC	6.83	<0.01	<0.01	0.59	0.58	7.65 ***	14.48 ***	<0.10	0.59
	GC–MS	6.51	<0.001	<0.001	0.48	0.36	4.66	11.17	<0.001	0.48
B6	HPLC	3.10 *	0.57 *	1.06	1.03	0.39	0.67	3.77 **	<0.01	1.03
	GC–MS	2.58	<0.001	1.02	0.96	<0.001	0.44	3.02	<0.01	0.96
B7	HPLC	1.26	0.22	1.02 ***	8.99 **	0.38	0.23	1.49	13.11 ***	22.10 ***
	GC–MS	1.06	<0.001	3.61	7.89	0.12	0.59	1.65	11.59	19.48
B8	HPLC	4.52 ***	2.56 ***	1.03 *	1.16	0.44	0.15 ***	4.67	<0.01	1.16
	GC–MS	3.57	<0.001	0.53	0.85	0.09	1.46	5.03	<0.001	0.85
B9	HPLC	2.71 ***	0.18	<0.01	0.44	0.29	0.16	2.87*	<0.01	0.44
	GC–MS	2.28	<0.001	<0.01	0.40	0.04	0.27	2.55	<0.001	0.40

Cannabidiol, CBD; cannabigerol, CBG; cannabinol, CBN; Δ9-tetrahydrocannabinol, Δ9-THC; cannabichromene, CBC; cannabidiolic acid, CBD-A; Δ9-tetrahydrocannabinolic acid, Δ9-THC-A. Overall, a higher content of some cannabionoids was determined by HPLC than in GC–MS, in each sample (*** *p* < 0.001, ** *p* < 0.01, * *p* < 0.05; One-way ANOVA with Bonferroni’s post hoc test).

## Data Availability

Data is contained within the article.
